# The role of forelimb motor cortex areas in goal directed action in mice

**DOI:** 10.1038/s41598-017-15835-2

**Published:** 2017-11-17

**Authors:** Karin Morandell, Daniel Huber

**Affiliations:** Department of Basic Neurosciences, University of Geneva, Geneva, Switzerland

## Abstract

Mammalian motor cortex consists of several interconnected subregions thought to play distinct roles in voluntary movements, yet their specific role in decision making and execution is not completely elucidated. Here we used transient optogenetic inactivation of the caudal forelimb area (CFA) and rostral forelimb area (RFA) in mice as they performed a directional joystick task. Based on a vibrotactile cue applied to their forepaw, mice were trained to push or pull a joystick after a delay period. We found that choice and execution are temporally segregated processes. CFA and RFA were both essential during the stimulus delivery for correct choice and during the answer period for motor execution. Fine, distal motor deficits were restricted to CFA inactivation. Surprisingly, during the delay period neither area alone, but only combined inactivation was able to affect choice. Our findings suggest transient and partially distributed neural processing of choice and execution across different subregions of the motor cortex.

## Introduction

The ability to manipulate objects is a fundamental characteristic of primate^1,2^ and rodent behavior^3,4^ and is highly dependent on frontal cortex circuits. The motor cortex can be subdivided into primary and premotor areas^5–7^. Premotor areas are found to be active during movement preparation, whereas the primary motor cortex is more likely to be active during movement execution^8–13^. In the context of forelimb-related choice behaviors, decision or motor preparation activity has been found to occur in premotor areas^11,12^ and has been used to reliably predict the upcoming choice^11,14–16^. Premotor areas are therefore thought to be important for processing the upcoming choice or motor planning, whereas the primary motor cortex is thought to be a main driver during the execution of movement, leading to the idea of a hierarchical organization of the motor cortex.

In rodents, two frontal cortical regions are critically involved in controlling the contralateral forelimb: the rostral forelimb area (RFA) and the caudal forelimb area (CFA). They have been identified by electrical^17–20^ and optogenetic microstimulation^21,22^, as well as by anatomical tracings^23–25^, yet their respective functional role is still unclear. It has been speculated that they are organized in an analogous way to the primate frontal cortex^17,19,23,26–28^ with CFA as the primary motor cortex and RFA as a premotor area.

However, the idea of a hierarchical premotor-motor organization is frequently challenged^5,22,29^. The arguments are based on the following observations: (1) Descending corticospinal neurons are not restricted to the primary motor cortex, but can also be found in neighboring premotor and somatosensory areas in primates^5^ and rodents^26^. (2) Cortical microstimulation of premotor areas triggers movement execution, including grasping or reaching in primates^30,31^ and rodents^17–19,22,27,32,33^. (3) The type of movement deficits caused by loss-of-function experiments in the rodent motor cortex have been found to be highly variable^34,35^, which make unclear to what extent the motor or premotor areas are necessary for movement choice or its execution. For example, lesions, pharmacological inactivation, cooling or optogenetic silencing of rodent motor cortex can halt^34^ or alter movements of the contralateral forelimb^19,36–40^, or leave movements unaffected^35^. (4) Finally, neuronal recordings during motor tasks failed to show different CFA and RFA activity in rodents^41–44^, suggesting that the two could be involved in similar neural processes.

To gain a better insight into the role of these two motor cortical areas in mice, we developed a two-choice forelimb joystick task in which the motor action was separated from the sensory stimulation by a delay period. CFA and RFA of trained mice were transiently silenced with optogenetic inactivation during different task phases to study their relation to the preparation and execution of a learned sensory motor transformation. If the rodent motor areas are organized analogously to the primate premotor and primary motor areas, we would expect that silencing RFA should affect choice and that silencing CFA would affect motor execution. In contrast, if rodent motor cortex is organized in a more parallel or redundant fashion where choice and execution of a motor plan are distributed among both areas, then combined silencing should affect these functions. Our results reveal the existence of both types of organizations and suggest that both areas are involved in processing choice and execution in a transient and partially distributed manner.

## Results

### Head-fixed mice can learn delayed response joystick task

**Figure 1:**
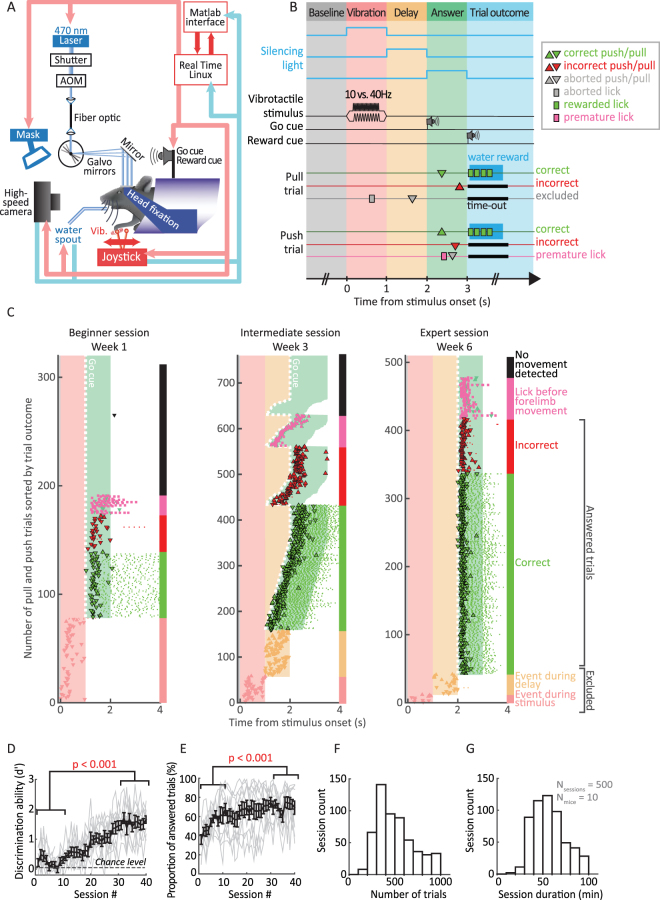
A delayed response task to investigate motor cortex function in head-fixed mice. (**A**) Head-fixed mouse in the automated behavioral setup. A vibrotactile stimulus (Vib.) was delivered through the joystick, indicating to push or to pull. Correct movements were rewarded with a drop of water and signaled by a sound cue. A laser beam was directed via a pair of galvanometric mirrors onto the skull and modulated by an acoustic optical modulator (AOM) and a shutter. Laser light was visually masked by a blue LED (Mask). Two high-speed cameras recorded a side- and top-view of the mouse. A real-time behavioral system controlled and recorded events. (**B**) Timeline of events during a trial. After a baseline period (0.5–2 s, grey background) during which mice were required to refrain from licking or moving the joystick, a vibration of high or low frequency was applied to the forepaw (1 s, light red background). The vibration was followed by a delay (1 s, orange background). The end of the delay was indicated by an auditory “go cue”. During the answer period (green background), mice were given 1 s to pull (downwards triangles) or push (upwards triangles) the joystick. Correct joystick movements (green triangles) were signalled by an auditory reward cue and a droplet of sweet water. Incorrect (red triangles) and anticipated events (grey markers) resulted in a 1 to 3 s time-out. Optogenetic inactivation was limited to the stimulus, delay or answer period. (**C**) Example learning session raster plots at different performance levels (beginner, intermediate, expert). In intermediate sessions the delay was gradually increased across trials. The trials are sorted by trial outcome. The vertical colored bar on the right of the raster refers to the outcome of each trial. Colors and symbols as in (**B**). (**D** and **E**) Learning curves showing the discrimination ability d’ (**D**) and proportion of answered trials (correct and incorrect trials, **E**) across learning sessions. Black error bars represent the mean ± SEM of 10 animals. Fine grey lines: individual mouse performance. The difference in performance between early (1 to 10) and late sessions (31 to 40) was statistically tested by using a paired t test on animal averages. (**F** and **G**) Histograms showing average number of trials completed per learning session (**F**) and session duration (**G**) for the 10 trained mice.

To study goal directed forelimb movements in the head-fixed mouse, we developed a forelimb based delayed response task. Mice were trained to push (“push trials”) or pull (“pull trials”) a miniature joystick in response to a high or low frequency vibration applied to the forepaw (Fig. 1A,B, Movie). Sensation and movement execution were temporally separated by a delay period (Fig. 1B, see Methods). After a baseline period (0.5 to 2 s), a vibrotactile stimulus (10 or 40 Hz) was delivered through the joystick specifying which movement direction would be rewarded (vibration period). After a subsequent delay period (1 s), an auditory “go cue” indicated the mouse to respond (answer period) by moving the joystick. Correct response trials (correct direction of the joystick movement) were rewarded with a drop of sweet water and signaled by an auditory cue. Incorrect responses (incorrect direction of the joystick movement) and aborted trials (licking or movement initiation before the go cue) were not rewarded and were followed by a time-out (Fig. 1B).

During training, task difficulty was progressively increased (Fig. 1C, intermediate session, Methods) and correct movement choice was computed with an adapted version of the sensitivity index d’ (see Methods), measuring the ability to discriminate between trial types. All mice learned to perform the task (d’ >1, Fig. 1D, i.e. >70% correct trials, Fig. 1–1). Mice reached criterion performance in 27 ± 10 (mean ± SEM) learning sessions (Fig. 1D), ranging from 19 to 49 sessions. The proportion of answered trials (correct or incorrect) increased with training and plateaued at 70% after 25 ± 6 (mean ± SEM) sessions (Fig. 1E). Mice performed 498 ± 8 (mean ± SEM) trials in daily sessions lasting 57 ± 0.7 minutes (mean ± SEM, Fig. 1F,G).

### Choice and execution are temporally segregated processes

**Figure 2:**
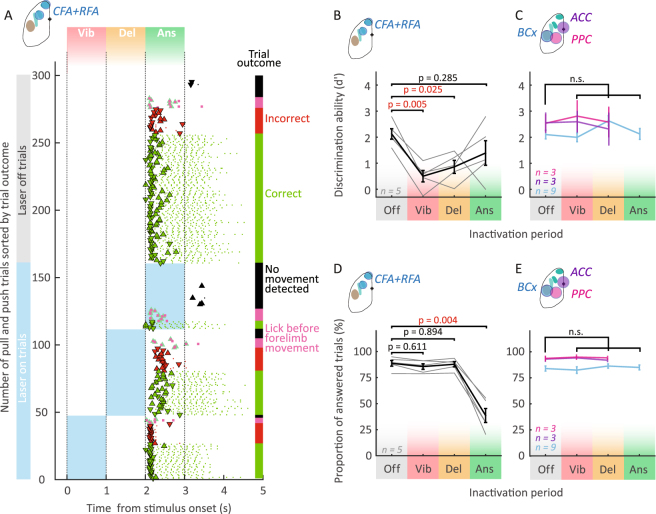
Forelimb motor cortex is necessary for choosing and executing joystick movements. (**A**) Example inactivation session raster sorted by trial outcome and inactivation condition (top, control trials; bottom, inactivated trials). The abscissa represents time from stimulus onset. Blue rectangles represent silencing period. In this example, CFA and RFA were silenced simultaneously. Markers (triangles for joystick events and dots for lick events) and vertical bars’ color code refers to the trial outcome (same than Figs 1 and 2). Note that silencing motor cortex early (vibration and delay period) changed mice’ joystick direction choice, while inactivation during the answer period didn’t affects execution of joystick movements. (**B** to **E**) Comparison of performance in control trials with performance in different inactivation conditions. Simultaneous inactivation of RFA and CFA (**B**,**D**) decreased the discrimination ability (**B**) and proportion of answered trials (**D**) while silencing other cortical areas (**C** and **E**, e.g. the barrel cortex (BCx), the anterior cingulate cortex (ACC) and the posterior parietal cortex (PPC)) did not change the performance compared to control trials (**C**,**E**). (**B**,**D**) Black error bars represent the mean ± SEM of 5 animals. Individual performances of mice: fine grey lines. (**C**,**E**) Colored error bars represent the mean ± SEM performance for control trials and silenced trials (purple, ACC; blue, BCx; magenta, PPC). For tests of significance for this and all other figures see Methods and Table 1.

After mice reached criterion performance, we tested if and at what moment CFA and RFA (contralateral to the “active” paw, Methods) are involved in the different aspects of the behavioral task. To perform transcranial optogenetic silencing of the contralateral forelimb motor cortex areas we used transgenic mice expressing channelrhodopsin-2 in cortical inhibitory interneurons (VGAT-ChR2-EYFP)^45^ in combination with a blue light (473 nm) laser scanning system (Fig. 1A, Methods). The laser light reached the brain through a translucent head cap and the intact skull^45^. A pseudo-random set of 1/3 of the trials was used for silencing (n = 9 mice, Methods). We first assessed if CFA and RFA are involved in the task by simultaneously inactivating both areas (Fig. 2A,B,D, Methods). When we optogenetically silenced these areas during the vibration or the delay period, mice made significantly more incorrect joystick movements compared to control trials (Fig. 2A,B, vibration period: d’_Vib_ = 0.5 ± 0.2, mean ± SEM, p = 0.005, delay period: d’_Del_ = 0.9 ± 0.2, p = 0.025, n = 5), but the probability of producing a motor response was not affected (Fig. 2D). In contrast, simultaneous inactivation during the answer period significantly impaired motor execution, reducing the number of responded trials to 38.6 ± 6% (Fig. 2D, mean ± SEM, p_Ans_ = 0.004, n = 5). In the remaining inactivated trials, in which mice overcame the inactivation effect, mice performed shorter joystick movements (Figs 2–1A,B, Fig. 2–2A,B) and at a slower speed (Figs 2–1C,D, Fig. 2–2C,D) without affecting movement onset (Figs 2–1E,F, Fig. 2–2E,F). Interestingly they produced, on average, the correct motor responses (Fig. 2B, d’_Ans_ = 1.4 ± 0.4 compared to d’_Off_ = 2.1 ± 0.2, p = 0.285). Taken together, these results suggest a double dissociation during different phases of motor tasks: before the “go cue”, motor cortex is necessary for choosing or preparing the appropriate motor plan (without affecting the drive for the upcoming motor output), while after the “go cue”, cortical drive is important for executing the movement (but no more for selecting appropriate motor plan).

**Figure 3:**
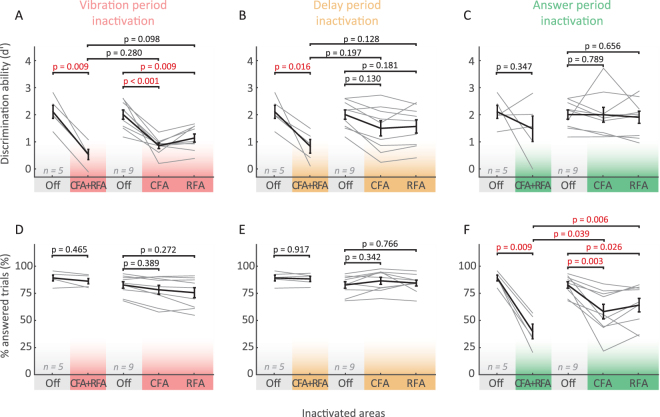
The specific role of CFA and RFA depends on the task period. Comparison between performance in control trials (e.g. Off, grey) and trials silenced on single areas at different periods: vibration period (red, **A** and **D**), delay period (orange, **B** and **E**) and answer period (green, **C** and **F**). Black error bars represent the mean ± SEM of the discrimination ability (**A** to **C**) and proportion of answered trials (**D** to **F**). Fine grey lines: individual mouse performance. Only combined inactivation (CFA and RFA) results in a significant decrease in performance in the delay period.

Is this effect specific to forelimb related motor areas or does the perturbation of neighboring cortical activity cause similar effects? We silenced three neighboring cortical areas: the anterior cingulate cortex (ACC), the posterior parietal cortex (PPC) and the barrel cortex (BCx). In rodents, ACC has been involved in effort based decision making^46,47^ and inactivation of ACC could affect the behavior if different values are associated with both actions. PPC plays a role in sensory guided choice^15,48–50^. Finally, the barrel cortex was chosen as a neighboring control area because it is a major sensory input system in rodents and disturbing its activity could act as a salient distractor. We found that when silencing ACC, PPC or BCx, neither d’ (Fig. 2C, see Table 1 for p values) nor the proportion of answered trials (Fig. 2E, see Table 1 for p values) were affected. This suggests that the neuronal activity in neighboring areas such as the ACC, PPC or BCx only plays a minor role during our behavioral task. Since RFA is also partially overlapping with a region termed ALM (anterior lateral motor cortex) which has been shown to control directional licking behavior and tongue movements^22,45,51,52^, we also analyzed if the inactivation affected licking dynamics in our task. We found that the timing of the licks and the licking rate (see Methods) were not affected by the inactivation of RFA nor CFA (Figs 3–1), confirming the results of others^42^. These results confirm that that the frontal cortex areas CFA and RFA play an important role in the execution and preparation of movements, compared to neighboring cortical areas.

**Table 1:** p values for Fig. 2C,E. PA, Proportion of answered trials.

Inactivated region	Behavioral measure	Figure	p values			N	test	post hoc
			Vibration period	Delay period	Answer period			
ACC	d’	2C	p > 0.999	p > 0.999	no data	3	Kruskal-Wallis	Tukey-Kramer
	PA	2E	p = 0.988	p = 0.988				
PPC	d’	2C	p = 0.952	p = 0.988				
	PA	2E	p = 0.988	p = 0.822				
BCx	d’	2C	p > 0.999	p = 0.972	p = 0.933	9		
	PA	2E	p = 0.999	p = 0.999	p = 0.983

### The effects of single area inactivations

To dissect the roles of RFA and CFA individually, we inactivated them separately during all three phases of the task. Similarly to the combined inactivation, silencing either CFA or RFA during the vibration period led to significantly less correct responses (Fig. 3A, p(CFA)< 0.001, p(RFA) = 0.009, n = 9) while the proportion of answered trials was unchanged compared to control conditions (Fig. 3D, p(CFA) = 0.389, p(RFA) = 0.272, n = 9). Activity in CFA and RFA is therefore necessary during the vibration period for choosing the correct answer.

During the delay period, individual inactivation of CFA or RFA was not sufficient to affect choice (Fig. 3A, p(CFA) = 0.130, p(RFA) = 0.181, p(CFA + RFA) = 0.025, n = 9), in contrast to the combined inactivation which significantly decreased d’ (Figs 2B and 3B). Subsequent motor execution was not affected by single or combined inactivation during the delay period (Fig. 3E, PA(Off) = 84 ± 2.6%, PA(CFA) = 86.1 ± 2.8%, PA(RFA) = 83.5 ± 2.3%, PA(CFA + RFA) = 86.4 ± 1.7%; mean ± SEM, p(CFA) = 0.342, p(RFA) = 0.766, p(CFA + RFA) = 0.894, n = 9). Together, these results confirm that both CFA and RFA are involved in processing choice before the “go cue”, yet in different ways: while during the vibration period, either area is necessary for correct choice outcome, only combined inactivation affects choice during the delay period.

Finally, we analyzed how inactivating CFA or RFA during the answer period affected choice and movement execution. We found that silencing either CFA or RFA significantly decreased the proportion of answered trials (Fig. 3F, CFA: 83.6 ± 2.8% to 58.2 ± 6.9%, p(CFA) = 0.003, RFA: 66.0 ± 6.3%, p(RFA) = 0.026, n = 9), yet the faction of correct choices remained the same (Fig. 3C). Taken together, after the “go cue” CFA and RFA therefore seem to be both involved in initiating or controlling the movement of the forepaw, while choice can no longer be affected.

**Figure 4:**
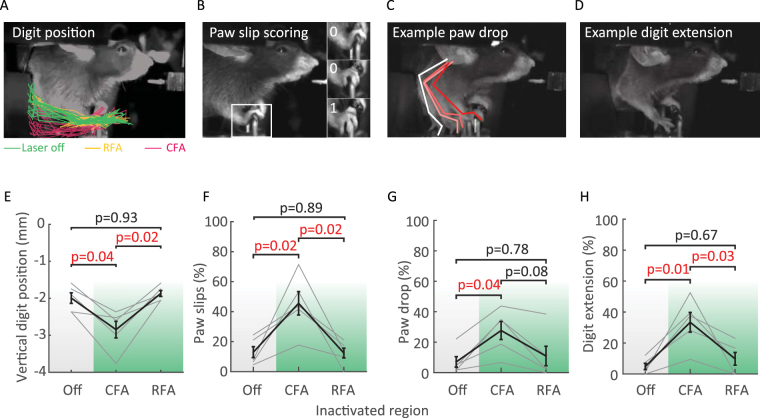
CFA inactivation causes fine motor deficits. (**A** to **D**) Profile views of head-fixed mice illustrating fine movement impairments: digit trajectories (**A**), paw “slips” (**B**), paw drops (**C**) and digit extension (**D**). **A**, Digit trajectories from the go cue to the end of the answer period. The paw position was quantified by tracking the vertical position of digits relative to the baseline position of the joystick. (**B**) Paw “slips” were scored if two digits laterally slid off the joystick, as illustrated on the zoomed paw bottom image. (**C**) Example paw drop. Paw drops were counted when the paw dropped below the handle of the joystick. (**D**) Example digit extension (paw opened and digits spread). (**E** to **H**) Population data of changes in paw closure in control (Off) and inactivated trials (CFA or RFA during the answer period, n = 5 mice). Black error bars represent the mean ± SEM of the measured movement impairment. Fine grey lines: values of individual mice. Note that CFA inactivation is sufficient to cause paw closure deficits while RFA is not.

However, indirect measurements such as the response rate or joystick trajectories might not be sufficient to capture fine differences between CFA and RFA inactivation. In fact, deficits in dexterity or digit closure have been observed after cortical lesions in rodents^4^ and primates^2^. We therefore carried out detailed analysis of the forepaw movement using videography. We found that paw movements were prevented in 20.3 ± 5.0% in CFA inactivated trials and to 8.9 ± 3.6% in RFA inactivated trials compared to only 5.0 ± 1.5% in control trials. In the remaining trials (in which the paw still moved) subtle, yet significant changes in the forepaw trajectory and closure were specific to CFA inactivation (Fig. 4), suggesting a distinct role of CFA in the control of more distal forelimb movements.

**Figure 5:**
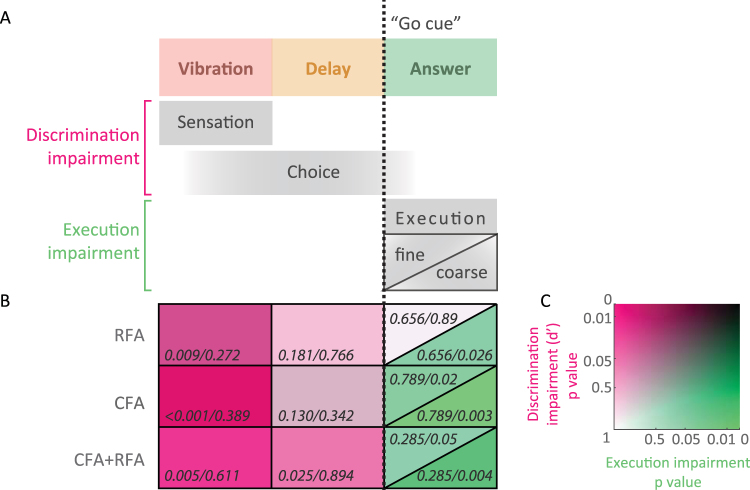
Summary of CFA and RFA inactivation. (**A**) Temporal distribution of putative cortical functions. D-prime was chosen as a measure of discrimination ability describing the process through which the presentation of a sensory stimulus ultimately leads to one of two movements, while coarse and fine motor execution were estimated by the proportion of answered trials and the proportion of paw “slips”, respectively. (**B**) Inactivation results summary matrix. Columns correspond to inactivation periods (vibration, delay and answer) and rows to inactivation locations (simultaneous CFA and RFA, CFA alone and RFA alone inactivation). The color code is explained in panel C. Note that discrimination ability and execution are sequentially affected. The ability to choose or plan the appropriate movement is mostly impaired when inactivating during the sensation and delay period (pink) while execution was only affected when inactivation was triggered during the movement (green). Also note that discrimination was most significantly impaired when CFA and RFA were silenced simultaneously during the delay period while inactivating the different forelimb areas individually showed similar non-significant weaker effects. Finally, the fine digit movements were altered by CFA inactivation only (% “slips” but also see Fig. 4) while overall joystick movement execution was not differentially affected by CFA or RFA inactivation (see Figs 2–1 and 3F). (**C**) Color code used in (**B**). Axes represent p values for comparison between control and inactivated trials. The y axis represents the discrimination ability (d’ p value from Fig. 3) and the x axis represents the p values for coarse and fine motor deficits measured by the proportion of answered trials (PA p value from Fig. 3) and the proportion of “slips” (p value from Fig. 4F), respectively. Axes are in a logarithmic scale.

Taken together, our results suggest first, that there is a switch in neuronal processing at the moment of the “go cue”, where correct choice can only be affected by manipulating neuronal activity before, and motor execution only after the beginning of the answer period (Figs 2, 3 and 5). Second, CFA and RFA are both involved in movement execution (Fig. 3F), yet fine, distal motor deficits are specific to CFA inactivation (Figs 4 and 5). Finally, correct choice (Figs 3 and 5) and many coarse aspects of movement kinematics (Figs 2–1, 2–2 and 5) are only affected by combined inactivation, indicating that there might exist considerable functional redundancy across CFA and RFA.

## Discussion

### A novel forelimb task for mice

Here we studied the role of different frontal cortical areas in a novel delayed joystick movement task for head-fixed mice. Using forepaw movements instead of licking^15,45,51,53^ as motor output, has several advantages. Although licking is a motor modality that facilitates training, it complicates the interpretation of neuronal data because it is performed instinctively by mice and is concomitant to reward consumption. Furthermore, to what extent insights gained from licking circuits can be extended to understanding the selection and execution of other goal directed actions, such as forelimb movements is unclear. Therefore, forelimb specific tasks for head-fixed rodents involving a manipulandum or a “joystick” are desirable and have started recently to emerge^39,42,44,54,55^. The most closely related behavioral paradigms to our study consist of rodents moving a lever spontaneously or upon a cue presentation to obtain a reward^39,42,44,54,55^. In these tasks, rodents are not required to select one of two actions, limiting the possibility of studying different motor plans.

Inspired by primate work^11,13,56–58^, we also separated stimulus from action with a delay period, thus allowing to independently investigate functions such as choice (or movement planning), execution and reward consumption. In our study, we have used “choice” as an umbrella-term (quantified by d’) to refer to the process through which the presentation of a sensory cue ultimately leads to the execution of one of two possible movements to obtain a reward (see Discussion in ref.^59^). An important aspect of the task was that the time required to be learned was similar, if not shorter, to comparable tasks in primates^9,13^. Also, our task enables mice to perform a high number of trials per session (498 ± 8 total trials per session, Fig. 1F), which is desirable for reliably interpreting behavioral results^59^. The symmetric, two-choice task design also makes the performance less sensitive to fluctuations in the engagement of the subjects^59^. Finally, head-fixation allows precise control of the environment facilitating the behavioral readout of fine movement deficits (Fig. 4) or the observation of subtle postural adjustments (see Discussion^15,60^) and makes the task suitable for neurophysiology experiments (e.g. two-photon imaging or electrophysiological recordings).

### Inactivation strategy and effects

Studies comparing the relative contribution of RFA and CFA to forelimb movements are still rare and use different loss-of-function strategies^19,34,37,61^. Here, we used optogenetic inactivation to precisely and transiently target different areas of the dorsal cortex in a non invasive manner. The optimal silencing protocol should completely silence the targeted area without affecting the surrounding areas. However, CFA and RFA differ in thickness and size^33,62^ and incomplete silencing could non significantly affect behavior. Our silencing approach is therefore a trade-off between spatial resolution and significance of behavioral effects. We chose 9 mW as inactivation power of all single areas (CFA, RFA, PPC, ACC, BCx) to affect similar amount of cortical tissue and ensure sufficient coverage of CFA to obtain a significant behavioral effect (Figs 1–3D–F). Silencing single areas with lower power (Figs 1–3E a 3 mW, p = 0.223) and silencing of areas surrounding the putative location of CFA (Figs 1–3F, p = 0.15) did not lead to significant effects. The lower efficacy in affecting behavior with moderate power might be due to the remaining active neurons of the CFA network (Figs 1–3C). For the combined inactivation of CFA and RFA, the laser was switched between the two sites (see Methods). The laser power was set to 3 mW per site to achieve higher spatial specificity and ensure minimal overlap between CFA and RFA inactivation areas. Using this approach, we found that even at 3 mW per site, the combined inactivation of RFA and CFA significantly affected behavior. In contrast, three times more power did not affect the behavioral performance consistently during the delay period (Fig. 3B). In general, combined silencing effects at lower power were in most cases stronger than single site inactivation (Table 2). This could imply that the specific combination of CFA and RFA (even at reduced laser power), and not just the extended inactivation are sufficient to produce the behavioral effects.

**Table 2:** Comparison between double and single area inactivation. p values of the difference in differences analysis are also reported directly on figures.

Figure #	Measure	Inactivation period	Region 1	Region 2	p value
2-1B	movement amplitude	answer	CFA + RFA	CFA	0.054
2-1B	movement amplitude	answer	CFA + RFA	RFA	0.049
2-1D	movement speed	answer	CFA + RFA	CFA	0.043
2-1D	movement speed	answer	CFA + RFA	RFA	0.022
3A	d-prime	vibration	CFA + RFA	CFA	0.280
3A	d-prime	vibration	CFA + RFA	RFA	0.098
3B	d-prime	delay	CFA + RFA	CFA	0.197
3B	d-prime	delay	CFA + RFA	RFA	0.128
3F	percent answered	answer	CFA + RFA	CFA	0.039
3F	percent answered	answer	CFA + RFA	RFA	0.006
3-3A	percent correct	vibration	CFA + RFA	CFA	0.451
3-3A	percent correct	vibration	CFA + RFA	RFA	0.134
3-3B	percent correct	delay	CFA + RFA	CFA	0.158
3-3B	percent correct	delay	CFA + RFA	RFA	0.100

### Distinct roles of motor cortical areas

The role of the motor cortex in the context of controlling goal directed limb movements is still debated, partly because several levels of deficits have been described in inactivation studies. Movements were found to be unaffected^35,40^ after cortical lesion and altered^4,19,37,39,40^ or completely halted^34,51,63^ upon motor cortex inactivation. This diversity of effects might be due to differences in task structure, loss-of-function methods or brain regions. Furthermore, neuronal activity recorded in CFA and RFA in behaving rodents^41,42,44^ encoded similar information, although CFA neurons seem to discharge after RFA neurons in a phasic short burst during forelimb tasks^64^. In our study, we have combined a novel task with a rapid and spatially restricted inactivation method for a finer dissection of frontal cortex function. We found that CFA and RFA (Fig. 2B,D) were involved in the completion of the task, in contrast to surrounding areas (Fig. 2C,E). During the answer period, inactivation of either area significantly disrupted motor execution, whereas choice was not affected (Figs 2D and 3C,F). Vice-versa, choice was only significantly affected during vibration and delay period (Fig. 2A–C). This implies that there is a change in function, switching from processing choice to motor execution at the time of the “go cue”. Such temporal specificity across the different phases of the behavioral task is difficult to reveal with other inactivation methods.

The effects of CFA or RFA inactivation on choice during the vibration period (Fig. 3A) also illustrate the rapid emergence of a motor plan during stimulus presentation. These results confirm observations from recent loss-of-function studies in delayed discrimination tasks^15,65,66^ where the choice of upcoming movements were altered when inactivation occurred during the stimulus presentation. However, our findings differ from a whisker based pole localization task, where choice was not affected during the sensory period inactivation (using lower laser power)^45^. The somatosensory forelimb area (SFA) and CFA are partially overlapping (Figs 1–3A and F ^33,42^,) and strongly interconnected^67^. The inactivation of SFA might therefore account for the increase in wrong choices during the sensory period. Nevertheless, the existence of trial type specific preparatory activity in CFA during the stimulus presentation suggests that CFA might participate to the discrimination between trial types^68^. On the other hand, inactivating RFA, which is more distant from the sensory representation, still significantly affected choice thereby highlighting the important role of motor areas in choosing the correct action.

Based on the CFA-RFA connectivity pattern^23–25,27,28^ and intracortical microstimulation studies^17–20,27,33^ CFA might be considered as primary motor area and RFA as a premotor area (however also see refs^19,34,44^). Indeed we found that fine movement execution deficits were specific to CFA inactivation (Fig. 4), confirming CFA lesion studies performed in rats^4,36,38^ and primary motor cortex in primates^1,69^. This result is in accordance with a rat cortical lesion study in which reaching skills and their recovery were more strongly impaired by CFA lesions than RFA, suggesting that CFA is more critical for skilled forelimb behavior^61^. However, movement initiation or coarse motor deficits were observed across both areas (Fig. 3). Whether these changes were caused by loss of motor control, loss of muscle tone^40,44^ or the change of sensory feedback (due to the close proximity to the sensory forelimb area, see Figs 1–3F), will have to be addressed in future experiments.

Whereas our results reveal an important role of CFA and RFA in sensory processing and motor control of the forelimb, other neighboring cortical areas, such as PPC^15,48–50^ or ACC^46,47^ have also been found to be involved in action selection in rodents. Our results do not provide evidence that either PPC or ACC play a major role in this task. Actually, recent studies suggest that the PPC network might be specifically involved in visual information processing during decision making^49,50^, while not necessarily recruited for maintaining the choice during a delay^15,70^.

### Distributed processing across motor areas

Our experiments also revealed differences in effects between the silencing of individual and combined areas (Table 2). For example, during the answer period behavioral performance (Fig. 3F) and specific movement kinematics (Figs 2–1B,D) were less strongly affected by individual area inactivation than combined inactivation, even at lower laser powers. Similarly, during the delay period, discrimination performance was robust to single CFA or RFA inactivation (Fig. 3B). The non significant or significantly lower effects during single location inactivation could be due to compensatory responses of other brain areas or a redundant role of CFA and RFA. Functional compensations have been described in several species. Studies in primates and rodents show that after a stroke in “primary” areas the partial recovery of the grasping ability can be lost after a transient pharmacological inactivation of premotor areas^2,69,71–73^. Furthermore, studies in primates and rodents show extensive reorganization of premotor areas after lesions or strokes in primary and secondary areas^18,61,71,72,74^. This plasticity also correlates with the degree of rehabilitation^18,63^ and it is suggested to be crucial for functional recovery of movements^2,61,71,74–76^. However, these compensation mechanisms take place over longer time scales. Determining whether fast compensatory mechanisms take place upon inactivation necessitate simultaneous silencing and neuronal recording experiments across multiple areas. Recently, Li *et al*. elegantly used this strategy and revealed interhemispheric compensation between anterior lateral motor cortices (ALM)^77^ in mice. Similar to the ALM areas, CFA and RFA are densely and directly interconnected^23,24,67^. In analogy, the weaker behavioral changes during single area inactivation might reflect compensation or redundancy between CFA and RFA.

Finally, transient manipulation of cortical circuits can potentially disrupt the activity of downstream or connected areas (e.g. basal ganglia or other cortical areas) needed to perform this task. Such “off-target” effects^78^ or diaschisis can only be identified by combining silencing with simultaneous neuronal recordings, allowing monitoring local effects as well as activity changes in downstream circuits.

In conclusion, we demonstrated that CFA and RFA are both involved in motor execution and selecting the appropriate motor plans. Individual inactivation of each area at different moments of the task suggest that both areas are recruited early during the sensory period participating in the sensory discrimination or leading to motor plan selection. During the delay period the behavior is relatively robust to single area inactivation, suggesting a distributed function among the motor system. Finally, during the execution phase both regions are necessary, while CFA has a specific role in fine movement control. Simultaneously recording and manipulating neuronal activity in forelimb areas in behaving animals will be necessary to determine if and how compensatory mechanisms can explain the observed behaviors.

## Methods

### Animals

Experiments were performed on 12 VGAT-ChR2-eYFP mice (B6.Cg-Tg(Slc32a1-COP4*H134R/EYFP)8Gfng/J, Jackson Laboratory, http://www.jax.org, RRID IMSR _JAX:014548) (see details in Tables 3 and 4). The animals were bred in hemizygous colonies and used for the experiments between 2 and 9 months of age. Mice from the three different litters were used. They were housed in single cages containing a cardboard house and bedding material, in a normal light cycle room that was dark from 7:00 P.M. to 7 A.M. Water reward based training and experiments occurred during the light phase. To motivate mice to engage in and learn the behavioral task, they were limited to 1 ml/day of water for ≥10 days before training^79^. On training days, mice received up to 1 mL of water or sweetened water (10% sucrose) during the experimental session. The consumed water volume was determined by weighing the mice before and after a session. If mice had gained less than 1 ml, they were complemented more than an hour later. Food was available ad libitum. The weight and health (posture, appearance of the fur and motor activity) were monitored daily. If mice did not maintain a sufficient body weight (below 80% of their original weight), additional water was provided. All procedures were carried out in accordance with the Institutional Animal Care and Use Committee of the University of Geneva and with permission of the cantonal authorities.

**Table 3:** Details about mice used for behavior and optogenetic experiments.

	Behavior mice										Mean ± SEM
	1116	1117	1119	1120	1130	1576	1577	1578	1579	1580	
Weight before water restriction (g)	24	23.5	23.8	25.1	25	27.8	28.6	26	28.7	26	25.8 ± 0.6
# of learning sessions	57	46	62	48	53	47	46	47	47	47	50.0 ± 1.7
# of photoinactivation sessions	32	55	8	56	46	35	42	33	34	40	38.1 ± 4.3

**Table 4:** Mice appearing in this paper.

Figures	Figure #	Behavior mice										Electrophys. mice	
		1116	1117	1119	1120	1130	1576	1577	1578	1579	1580	5227	5228
Learning raster	1C				X								
Learning parameters	1D-G	X	X	X	X	X	X	X	X	X	X		
Silencing raster	2A									X			
Performance is affected by CFA and RFA inactivation	2B + 2D						X	X	X	X	X		
Performance is not affected by inactivation of BCx	2C + E	X	X	X	X	X	X	X	X	X	X		
Performance is not affected by inactivation of ACC and PPC	2C + E		X		X	X							
Motor cortex function is temporally segregated but spatially redundant	3	X	X	X	X	X	X	X	X	X	X		
CFA inactivation causes fine motor deficits	4						X	X	X	X	X		
Summary of CFA and RFA inactivation	5	X	X	X	X	X	X	X	X	X	X		
Transcranial optogenetic silencing spatial properties	1-1						X	X	X	X	X	X	X
Detailed analysis of movement dynamics	2-1						X	X	X	X	X		
Distribution of movement kinematics	2-2						X	X	X	X	X		
Motor cortex inactivation effects are restricted to the silenced trial	2-3	X	X	X	X	X	X	X	X	X	X		
Licking is not affected by forelimb motor cortex inactivation	3-1	X	X	X	X	X	X	X	X	X	X		
Movies							X						

### Surgical procedure for transcranial optogenetic cortical silencing

For the purpose of head fixation and optical access, mice were implanted with a small titanium head post and the skull was covered with a transparent cap^45^. Mice were anesthetized with isoflurane (3% and 1.5% by volume in O2, for anesthesia induction and maintenance respectively; Isoflurane Attane, Provet AG) and mounted in a stereotaxic device equipped with a custom made heating pad to maintain the body temperature. Eyes were covered with Vaseline. Prior to the surgery, mice received anti-inflammatory (2.5 mg per kg body weight dexamethasone intramuscular), analgesic (0.1 mg/kg buprenorphine intramuscular, 5 mg/kg Carprofen subcutaneous) and local anesthetic drugs (1% lidocaine subcutaneous under the scalp). After removing the skin above the skull, a thin layer of cyanoacrylate glue was applied to the dried skull. The head post was cemented to the interparietal bone and the area over the skull was covered with a thin layer of transparent dental acrylic (Lang Dental). After curing, the dental acrylic was polished and covered with a layer of cyanoacrylate glue to smooth the surface and thereby render it transparent.

### Behavioral setup/Apparatus

To precisely control and record behavioral events, we used an automated behavioral control system (Fig. 1A)^53^. The behavioral setup was enclosed in a custom-made light and sound proof box. Mice were placed inside a horizontal aluminum tube (32 mm internal diameter) and head-fixed by the titanium head-bar with custom clamps located in front of the tube. The tube was cut to allow mice to freely move their forelimbs. For body support, mice placed their left forelimb on a static paw rest and their right forelimb on a movable joystick. The joystick was custom made from a 1.3 mm diameter rod connected to a rotary encoder (Miniature Absolute Magnetic Shaft Encoder MA3 12 bit, www.usdigital.com). Two pairs of repulsive magnets (S-04-1.5-N, supermagnete.ch) provided the spring load to the joystick. The vibrotactile stimuli (10 Hz or 40 Hz sinusoidal) were delivered to the base of the joystick using a speaker coil. The joystick position from the rotary encoder was decoded with a microcontroller (Arduino Due, www.arduino.cc) and recorded with an analog/digital acquisition board (acquisition rate 5 kHz, PCI-MIO-16E-4, National Instrument). The real-time control system was connected through Ethernet to a MATLAB (Mathworks, RRID:SCR _001622) user interface computer (more details in^53^). An electrical lickport was placed in front of the snout and licks were detected as binary events^80^. Liquid reward delivery was controlled by a solenoid valve (The Lee Company). High-speed video (60 frames per second) was acquired through USB cameras (Firefly MV USB Monochrome Camera, Point Grey) using custom MATLAB routines. Potential sounds produced by the vibrotactile stimuli were masked by white noise delivered during stimulus presentation. A “go” cue (“beep” sound) to signal the movement period onset and a “reward” cue (“click” sound) to signal a correct behavioral response were delivered by using a buzzer (5 kHz, 80 ms) and a speaker (single 20 ms square pulse), respectively.

### Behavior

#### Task structure

The trial structure was designed to separate stimulus presentation from action by a delay period (Fig. 1B). Two trial types were possible (“pull” and “push” trials). The trials started by a 0.5 to 2 s long baseline period in which mice were required not to move the joystick. The trial onset was indicated by turning on a blue mask LED (470 nm). A vibrotactile stimulus (10 or 40 Hz) was delivered through the joystick to the right forepaw for 1 second followed by a delay period of 1 second. The end of the delay period was indicated by an auditory “go cue” (buzzer 80 ms, 5 kHz) and informed the mice that they could pull or push the joystick to report their choice with their right paw. Answer period was 1 second long. If mice moved the joystick to the correct direction, a “click” sound notified the mice that a ∼4 uL sweet water droplet (10% sucrose) was available upon licking to restrict licking to reward periods. If mice moved before the go cue or licked before moving the joystick, the blue mask LED turned off, the mice were not rewarded and received a 2 to 3 s time-out before the next trial was started. The time-out was reinitialized if additional movements (joystick or licks) were detected. In order to prevent choice bias observed in similar behavioral paradigms^45,81^, the trials were chosen based on a fixed set of rules during training and inactivation experiments. The sequence of trial types was random except in the following two situations. First, if 3 consecutive trials were of the same type, the next trial type was switched. Second, if mice did not respond correctly to one trial type (more than 4–6 out of 9 trials), that trial type was repeated. This procedure resulted in a minimal bias towards the end of the training (−0.36 ± 4.82%, mean ± SEM). The bias was computed as percent correct in push trials minus percent correct in pull trials.

#### Behavioral training procedure

Mice were trained to learn the behavioral task progressively. The training started after 5 to 7 short daily handling sessions (5 to 10 minutes). The first 3 to 5 training sessions consisted in a head-fixed pre-training period designed to (1) habituate mice to the behavioral setup, (2) teach mice that water is delivered by the lickport, (3) associate the “click” sound (reward cue) with reward availability upon lick, (4) discriminate between the reward cue and the stimulus and (5) prevent mice to lick except during reward periods. The joystick was immobilized for this part of the training. The pre-training was structured as a Go/No-Go task. In No-Go trials, one of the two vibrotactile stimuli was delivered and mice were required to withhold licking behavior. Correct No Go trials increased the probability of Go trials. In Go trials, the reward cue sound would be concomitant with the delivery of a ∼10 uL % sucrose water drop (“learning click trials”), or a 1.5 s period in which the mouse could be rewarded only if it licked upon the reward cue (“testing click trials”). The proportion of “learning click trials” was decreased progressively as performance improved. After this training phase, mice associated the reward cue with reward availability upon lick and showed moderated spontaneous licking. In the next phase, mice were required to associate the vibrotactile stimuli with a joystick movement (pulling or pushing). The vibrotactile stimulus was followed by a 50 ms delay period, after which an auditory go cue signalled the end of the withholding period and that mice were allowed to pull or push the joystick. The duration of the delay period was progressively increased within the training session (by 50 ms at each correct trial and up to 1 s) and across learning sessions (initial delay duration was the mean delay of the previous session). Similarly, the required joystick movement amplitude to receive a reward was increased progressively (1 mm to 10 mm) within and across learning sessions. The final required movement amplitude for push or pull trials was 10 mm and sub-threshold movements were ignored. Premature (before the go cue) joystick movements or licking (before the reward cue) resulted in a 1 to 3 s time-out before the next trial. During the learning period, the trial type sequence was automatically chosen to correct for potential response bias.

### Transcranial optogenetic cortical silencing

Inactivation trials were randomly intermingled with control unperturbed trials with a probability of 30 to 50%. Light from a 473 nm laser (DHOM, DHL-100A) was controlled by an acousto-optical modulator (AOM; MTS110-A3-VIS, Quanta Tech) and a shutter (Vincent Associates) (Fig. 1A). The AOM provided analog control of laser intensity. Light exiting the AOM was injected into a single mode optical fiber (3.0 *μ*m @ 480 nm; P1-405B-FC-2; Thorlabs) and a 2D galvanometer scanning system (“scanner”, GVSM002, Thorlabs) via a lens system (PAF-X-11-PC-A and PAF-X-5-A; Thorlabs). The mirrors of the scanner, the shutter and the AOM were controlled by the real-time behavioral control system^45^. Light from the scanner passed through a 5x beam expander and was focused onto the skull surface with a f = 200 mm lens (AC508-200-A, Thorlabs). The laser had a Gaussian profile with a beam diameter of approximately 300 *μ*m. Laser power was checked on a daily basis using a handheld power meter (Lasercheck, Coherent). To inactivate motor cortical areas on the left hemisphere (contralateral to the right, active paw), the laser beam was directed to the target coordinates by the scanning mirrors and the power was modulated by an AOM. To perform simultaneous CFA and RFA inactivation, the beam was displaced between the two targets at a 40 Hz frequency and the laser was blanked with the AOM in between the targets. To deprive mice from visual cues originating from the inactivation laser, a “masking” light was turned on at the beginning of every trial using a 470 nm LED (Luxeon Star) close to the eyes of the mice and controlled by a driver circuit (Luxdrive, Buckpuck 3021). The masking light^82^ began 0.5 to 2 s before the stimulus period and lasted 5 s. The light was turned off simultaneously to the initiation of the time-out.

#### Stereotaxic coordinates

The coordinates for optogenetic silencing were based on published studies using intracortical tracers injection^24,67^, motor maps generated by intracortical microstimulation experiments^22,42^: CFA (ML = 1.8, AP = 0.8), RFA (ML = 0.9, AP = 2.7), BCx (ML = 3.5, AP = −1.5), ACC (ML = 0.15, AP = 0), PPC (ML = 1.7, AP = −2, in millimeter, ML: mediolateral, AP: anteroposterior, relative to bregma, contralateral hemisphere). To ensure that the stereotaxic coordinates from the literature were reliable, we used the location of the somatosensory representation of the forelimb (SFA) as a second reference point in addition to bregma. The coordinate of SFA were detected by performing intrinsic signal imaging through the transparent skull cap (see Methods^82^, data not shown).

#### Electrophysiological recordings for laser power calibration

To evaluate the spatial and functional properties of the transcranial optogenetic cortical silencing technique, we recorded neuronal activity in CFA while probing the surrounding areas with the 470 nm laser beam. Extracellular spikes were monitored using silicon probes (Neuronexus, A1 × 32–10 mm-50–413) under awake, non-behaving conditions in 2 mice. Action potentials were detected, amplified, multiplexed, digitized and recorded at 20 kHz with Open Ephys hardware and software (http://open-ephys.org). Mice were anaesthetized with isoflurane and a 1 mm craniotomy (2 mm lateral and 1 mm anterior to bregma) was performed to target CFA. Before lowering it into the brain, the silicon probe was covered with DiI (Thermofisher, V22885) to mark the location of the recording sites and was inserted with a 30° angle in the coronal plane. After the silicon probe was in place for 30 minutes, electrophysiological recording started and the laser illumination was triggered every 7 s for 1 s at different preset distances from the silicon probe. Optogenetic inactivation was achieved by the transmission of the laser light through the skull and translucent dental cement, like in the behavioral experiments. The power was varied randomly (1, 2, 3.5, 5 and 9 mW) at every location. The probed distances were 0, 0.5, 1, 1.5 and 2 mm from the recording location. The 2 mm distance condition corresponded to the location of RFA as illustrated in Fig. 1–1A. In total, we obtained 12.2 ± 0.9 repetitions per location and laser intensity for 32 units.

Extracellular recordings were bandpass filtered (300–3000 Hz). Spike detection and cluster classification was performed offline by using Wave _Clus (https://github.com/csn-le/wave_clus). We extracted 32 units from 2 animals. Units were then classified according to the response to laser illumination as enhanced (putative VGAT + , interneurons) or silenced units (putative VGAT-, pyramidal neurons). Movement and other artifacts detected as simultaneous electrical deflections on all the electrodes were used to exclude trials from the analysis. Effect of laser illumination of ongoing neuronal activity was quantified as “normalized firing rate” by dividing the spike rate during the first second of laser illumination to the firing rate of the preceding second. The recorded neuronal signals and unit classification were visually inspected for each trial.

#### Laser power calibration

Based on the electrophysiological calibration experiment (Figs 1–3A) and pilot behavioural data (Figs 1–3D–F) we chose 9 mW per site as default power for all single area inactivation experiments (CFA, RFA, ACC, PPC, BCx). At 9 mW, the average spiking activity dropped to 20.3 ± 5.3% compared to baseline, while affecting most of the surface area considered as CFA or RFA (Figs 1–3C)^33,42^, yet still allowing spatial specificity (Figs 1–3F). For combined inactivation experiments, the silencing power was 3 mW per site (6 mW in total) (see Discussion).

### Behavioral analysis

#### Joystick movement kinematics

To track the paw movement on the joystick, the rotary encoder signals were recorded at 5 kHz. Trajectory signals were filtered with a 4th-order Butterworth filter with a low pass cut-off frequency of 12 Hz and offset corrected by subtracting the average pre-stimulus period (baseline). Joystick trajectories were analyzed in a semi-automated manner using a custom MATLAB script and joystick kinematics were computed: amplitude (Figs 4–1A), movement speed (Figs 4–1C) and movement onset (Figs 4–1E) as illustrated.

#### Tracking and scoring of forelimb movements

To quantify fine paw movement impairments not captured by the joystick trajectory, in a subset of experiments (N = 5) we recorded high-speed videos (60 fps) and manually scored the forelimb movements during the execution of the task (Fig. 4). First, we tracked the position of the tip of the digit to reconstruct the paw trajectory in case mice released the joystick. We focused on three conditions: control trials, RFA and CFA inactivation during the answer period. In addition to paw trajectories, we observed other behavioral deficits that were classified and scored as follows: (1) paw “slip”, when more than two digits slipped laterally off the joystick (Fig. 4B,F); (2) paw “drop”, when the paw dropped below the handle of the joystick (Fig. 4C,G); (3) digit extension, when the paw was opened and digits spread (Fig. 4D,H). Scoring was done blind to trial type.

#### Performance analysis and statistics

Trials were classified according to their outcome: “correct” (correct supra-threshold joystick movement), “incorrect” (opposite supra-threshold joystick movement), “no movement detected” during the answer period and “anticipated lick” (licking before moving the joystick). Trials with premature events (threshold crossing prior to the go cue) were excluded from the analysis for the inactivation experiments. In a subset of sessions mice did not answer during the initial period of the session and mice typically stopped answering after satiation^53,79^. In order to analyse a homogeneous performance (without periods of inactivity due to external factors), periods at the beginning (up to the 5th correct trial) and at the end (last 5 correct trials) of the behavioral sessions were excluded from the analysis.

We quantified the discrimination ability using d-prime (d’, sensitivity index) rather than percent correct to take potential bias into account^59^. d’ was quantified as following: d’ = z (correct pull rate) + z (correct push rate), where z is the normal inverse of the cumulative distribution function. Only correct and incorrect trials were included to compute d’. Positive d’ values (maximum d’ = 4.65) resulted from a majority of correct pull and push trials; negative d’ (minimum d’ = −4.65) corresponds to a majority of incorrect trials while null values mean chance level. To exclude infinity values from the formula, null and equal to one correct rates were rounded to 0.001 and 0.999, respectively. The fraction of answered trials included correct and incorrect trials (Fig. 1C). Learning sessions with changes in performance due to rig modifications were excluded from the analysis. Behavioral changes caused by cortical inactivation were evaluated by comparing control trial and inactivated trial performances. A Kruskal-Wallis test was used to compare performance in control and different inactivation conditions after testing for normality and homoscedasticity using the Kolmogorov-Smirnov test and the Bartlett test, respectively. The Fisher’s least significant difference test was used as post-hoc unless stated otherwise. P values are reported on the figures or in Table 1. Difference in differences analysis (DID)^83^ using a multiple linear regression analysis was performed to evaluate the significance of the differential effect between combined (CFA + RFA) and single inactivation conditions (CFA or RFA). Normality was assessed using the Shapiro Wilk test and heteroscedasticity was corrected by using a robust regression^84^. All results are reported as mean ± SEM.

## Electronic supplementary material

Movie

Supplementary information
