# Supplementary material for: The role of forelimb motor cortex areas in goal directed action in mice
Source: Sci Rep. 2017 Nov 17;7:15759. doi: 10.1038/s41598-017-15835-2 (PMC5693936; doi:10.1038/s41598-017-15835-2)
Supplement: Supplementary file 2 — Supplementary information [file 41598_2017_15835_MOESM2_ESM.pdf]

# Supplementary figures : The role of forelimb motor cortex areas in goal directed action in mice

**Karin Morandell, Daniel Huber**

## Figure 1-1 : Percent correct increases with training

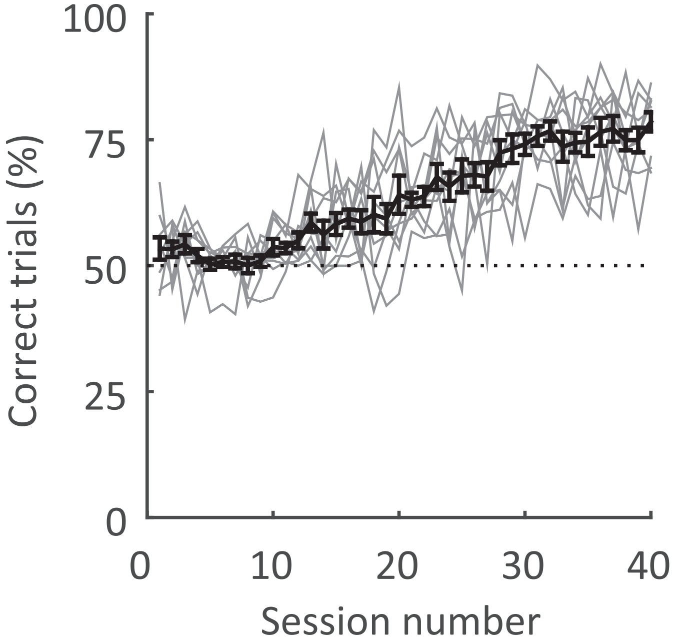


Learning curve showing increase of the proportion of correct trials across training sessions

## Figure 1-2 : Example learning sessions

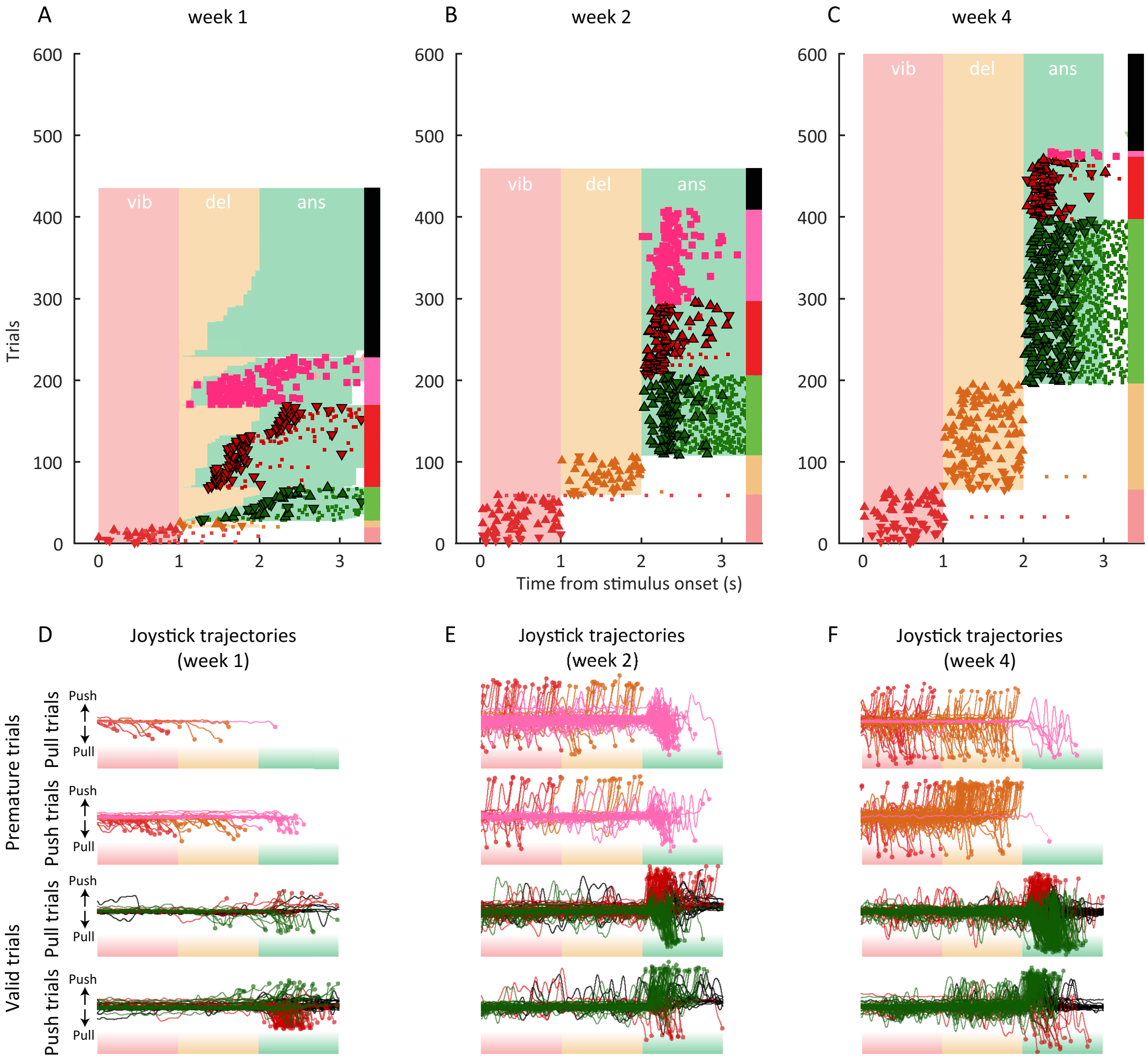


**A to C.** Example raster plots of learning sessions at different performance levels (beginner, intermediate and expert). Colors and symbols are the same as in figure 1C.

**D to F.** Joystick trajectories of example sessions illustrated above sorted by trial types (pull and push trials) and trial outcome (trials with premature events and valid trials). Color code is the same as in panels A to C. Trajectories are ended at point of detection (threshold crossing illustrated by circular marker). Note that joystick movement threshold is increased across sessions.

# Figure 1-3 : Transcranial optogenetic silencing spatial properties

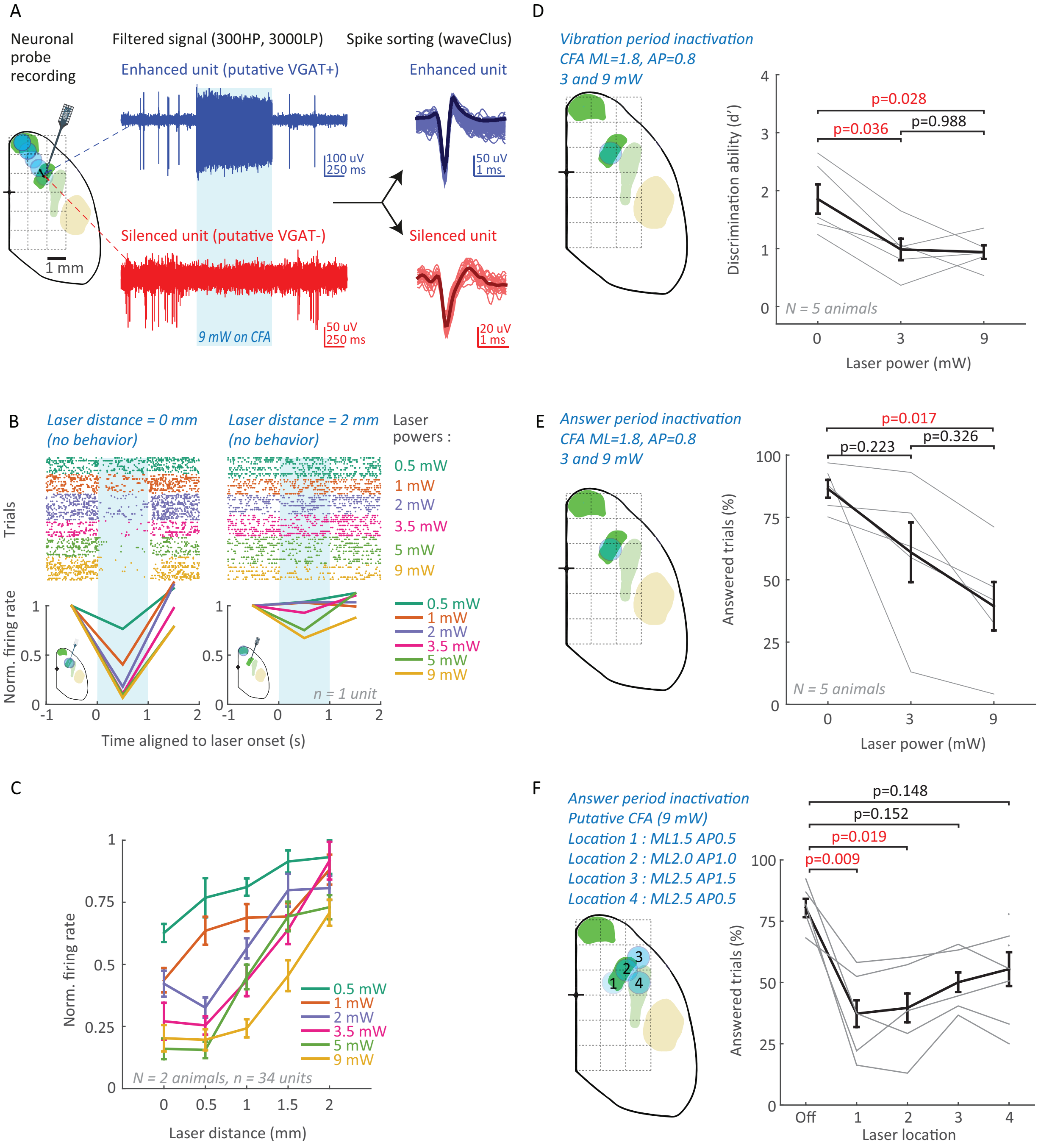


Legend on next page.

**A.** Experimental design for silicon probe neuronal recordings in awake non-behaving animals. *Left*, Schematic top view of mouse brain hemisphere. Shaded areas represent from top to bottom: RFA, CFA, SFA (sensory forelimb area) and barrel cortex. Silicon probe was inserted into the cortex: ML=2.0, AP=0.8. The laser beam was directed on CFA (ML=1.8, AP=0.8) or distanced of 0.5, 1, 1.5 or 2mm as illustrated. *Middle*, example filtered neuronal probe recording signal for an enhanced (*top*) and a silenced unit (*bottom*). Blue shading represents the "laser on" period. *Right*, example spike shapes obtained from spike clustering. Spikes were clustered (Wave clus) and sorted as enhanced (putative VGAT+ neuron) or silenced (putative VGAT- neuron) unit type based on the relative number of spikes during the "laser off" and the "laser on" period.

**B.** Example trials showing a silenced unit (putative pyramidal neurons) firing rate drop during laser inactivation in awake non-behaving animals. Each raster plot shows the behavior of the unit before, during and after laser illumination depending the distance and the power of the laser illumination. The distance between the recording site and the laser beam is 0mm or 2mm. The firing rate was computed for each epoch (-1 to 0, 0 to 1 and 1 to 2 s) and normalized to the pre laser period for each power (0.5, 1, 2, 3.5, 5 and 9 mW) and each distance (0, 0.5, 1, 1.5 or 2mm).

**C.** Silenced units firing rate for different laser distances and powers (N=2 animals, n=32 units) in awake non-behaving animals. D, Motor impairment as a function of putative CFA laser location (location 1: ML1.5 AP0.5, location 2: ML2.0 AP1.0, location 3: ML2.5 AP1.5, location 4: ML1.5 AP0.5). Note that the performance was significantly affected only for location 1 and 2.

**D.** Behavioral choice impairment (discrimination ability, d') as a function of laser power (3 and 9mW) while inactivating CFA during the vibration period. The performance was significantly affected at 3 mW and 9 mW.

**E.** Motor impairment (proportion of answered trials) as a function of laser power (3 and 9 mW) while inactivating CFA during the answer period in behaving animals. The performance was only affected at 9 mW.

**F.** Motor impairment as a function of putative CFA laser location (location 1: ML1.5 AP0.5, location 2: ML2.0 AP1.0, location 3: ML2.5 AP1.5, location 4: ML1.5 AP0.5). Note that the performance was significantly affected only for location 1 and 2.

## Figure 2-1 : Detailed analysis of movement dynamics

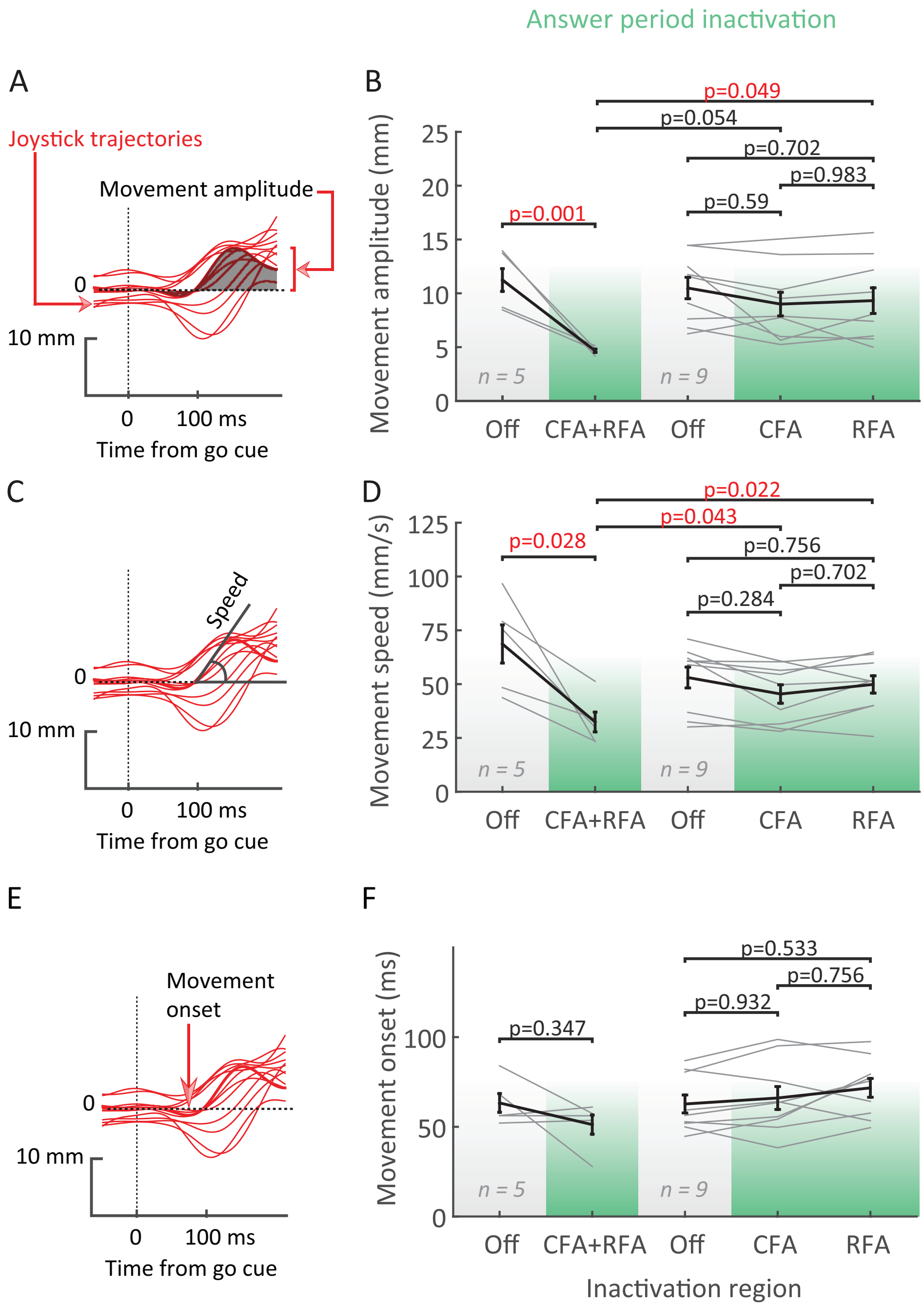


**A,C,E.** Example joystick trajectories illustrating the three measured movement kinematics : movement amplitude (peak, A), movement speed (slope, B) and movement onset (local maxima of first derivative preceding first peak on joystick trajectory, C). The y axis is in mm of displacement of the joystick.

**B,D,F.** Population data for measured movement kinematics. Black error bars represent the mean±SEM of the measured movement kinematic. Fine grey lines: values of individual mice. Note that only simultaneous CFA and RFA inactivation decreases movement amplitude and speed. In contrast, the movement onset is not delayed by motor cortex inactivation.

## Figure 2-2 : Distribution of movement kinematics

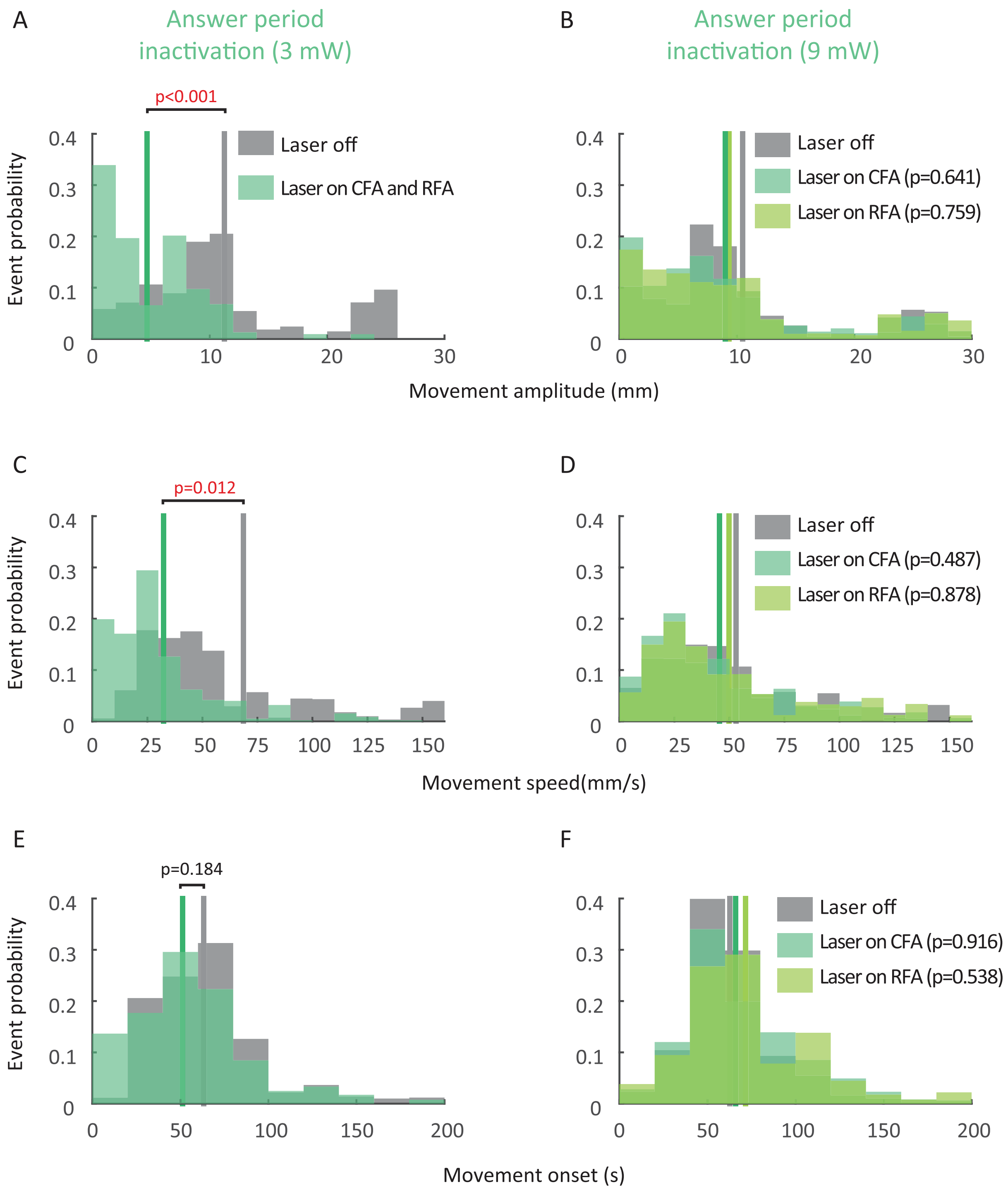


Movement kinematic distributions from figure 2-1 for combined (left, CFA and RFA) and single region inactivation (CFA or RFA). Simultaneous CFA and RFA inactivation strongly decreases movement amplitude and speed of the joystick. In contrast, joystick movement onset was not delayed by inactivation.

## Figure 2-3 : Motor cortex inactivation effects are restricted to the silenced trial

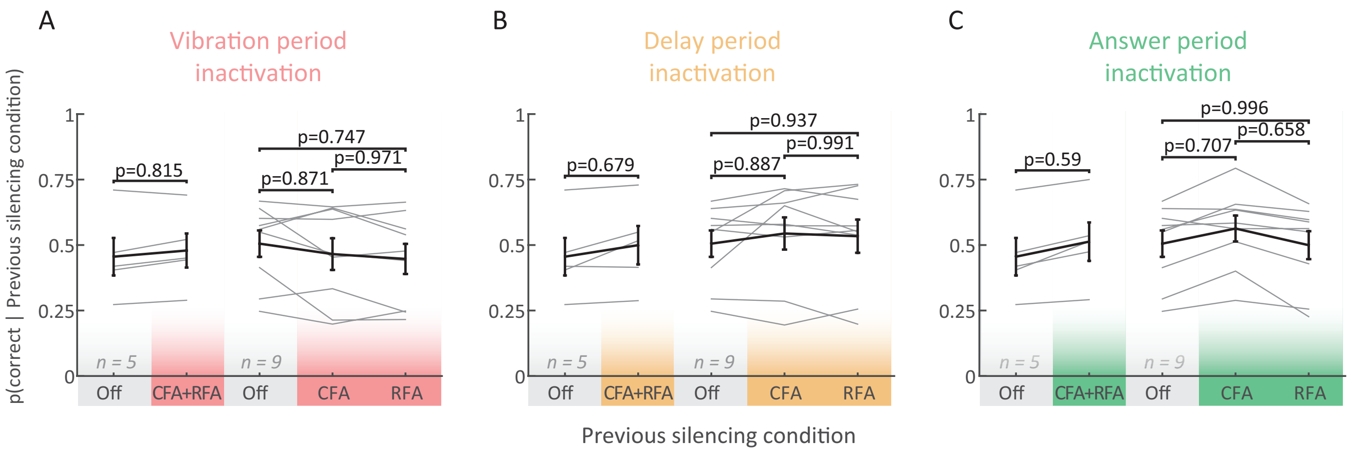


Measurement of the temporal effect of inactivation by computing the probability of a control trials (laser off) being correct given the previous trial inactivation condition (abscissa). All trials (including trials with premature aborted movements) are included for this analysis. Regardless of the inactivation condition of the previous trial, the correct trial probability is not different in control trials following control or silenced trials.

**A.** Probability of correct control trial given that the previous trial was a control trial (Off) or silenced during the vibration period simultaneously on CFA and RFA (CFA+RFA), CFA or RFA.

**B.** Same than in A but previous trial silenced during the delay period.

**C.** Same than in A but previous trial silenced during the answer period.

**Figure 2-4 : Decrease in proportion of correct trials is restricted to motor cortex inactivation**

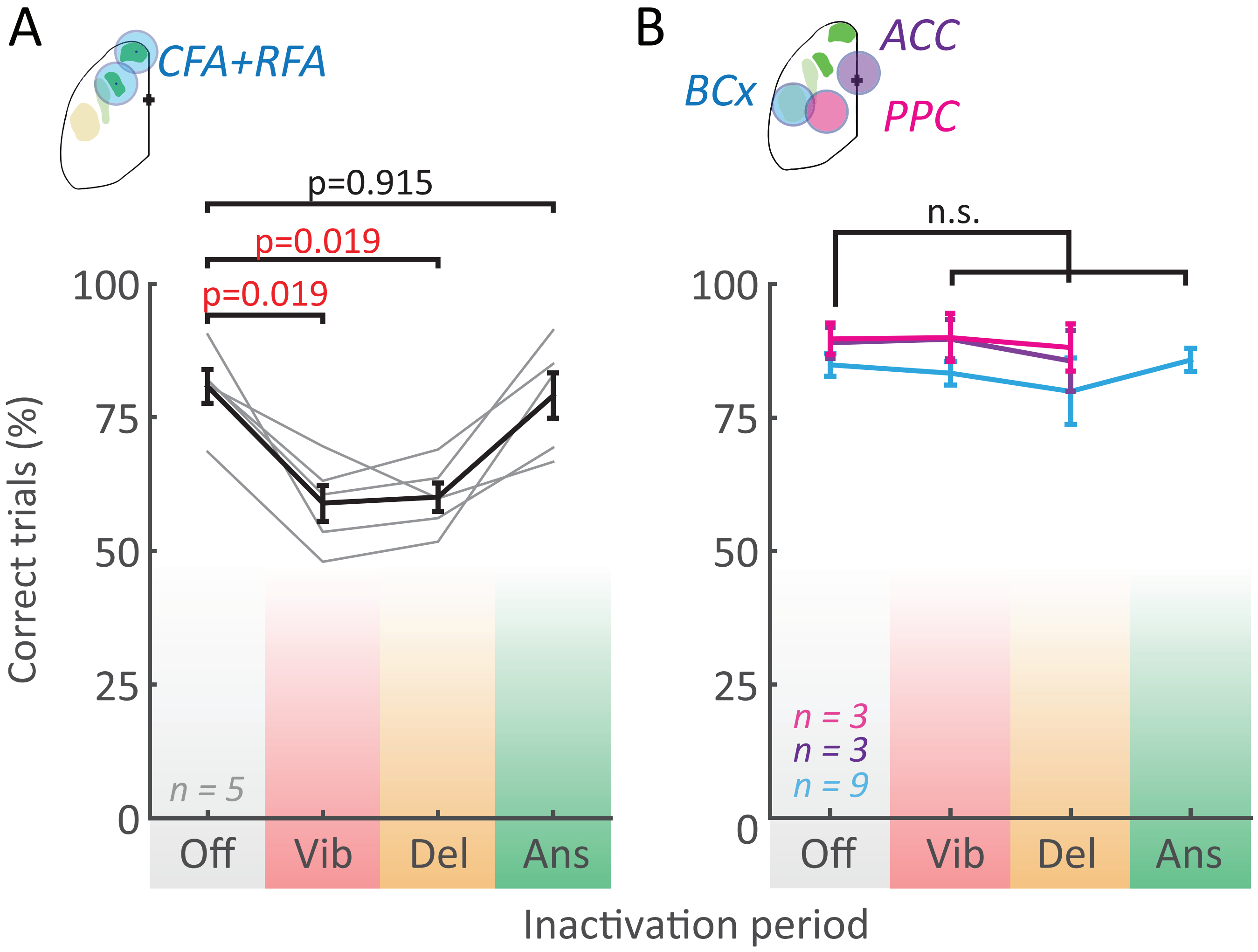


Performance in control trials and in different inactivation conditions. Simultaneous inactivation of RFA and CFA (A) decreased the proportion of correct trials among correct and incorrect trials while silencing other cortical areas (B, e.g. the barrel cortex (BCx), the anterior cingulate cortex (ACC) and the posterior parietal cortex (PPC)) did not change the performance compared to control trials (B). Black error bars represent the mean±SEM of n animals. Individual mice' performances are shown as fine grey lines. (B) Colored error bars represent the mean ± SEM performance for control trials and silenced trials (purple, ACC ; blue, BCx ; magenta, PPC). For tests of significance for this and all other figures see Methods and table 3.

## Figure 3-1 : Licking is not affected by forelimb motor cortex inactivation

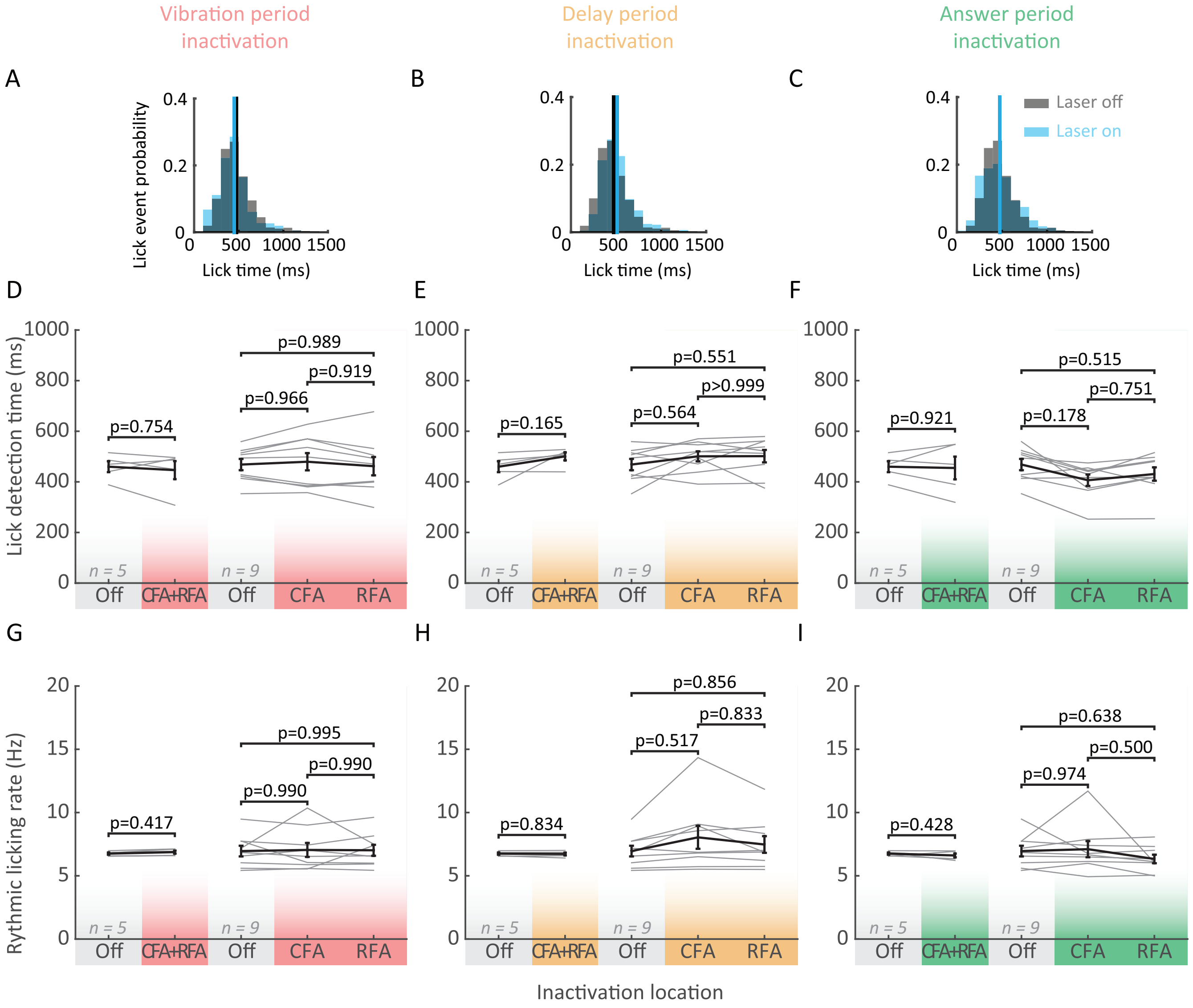


**A to C.** Histograms showing lick times distribution in control trials (grey) and trials inactivated at different periods: vibration period (A), delay period (B) and answer period (C). Note that there is no difference between laser off (grey) and laser on (blue) lick times. Vertical lines represent median of all lick times for 5 animals. For this example, CFA and RFA was inactivated simultaneously.

**D to I.** Lick timing analysis on population data. Lick detection time (D to F) and rhythmic licking rate (G to I) in different inactivation periods (vibration (D and G), delay (E and H) or answer period (F and I)) and locations. Black error bars represent the mean±SE of the lick time and licking frequency. Individual mice' mean measures are shown as fine grey lines. For tests of significance for this and all other figures see Methods. Note that licking is not affected in any of the inactivation conditions compared to control conditions.

## Figure 3-2 : Motor cortex silencing decreases the proportion of correct trials

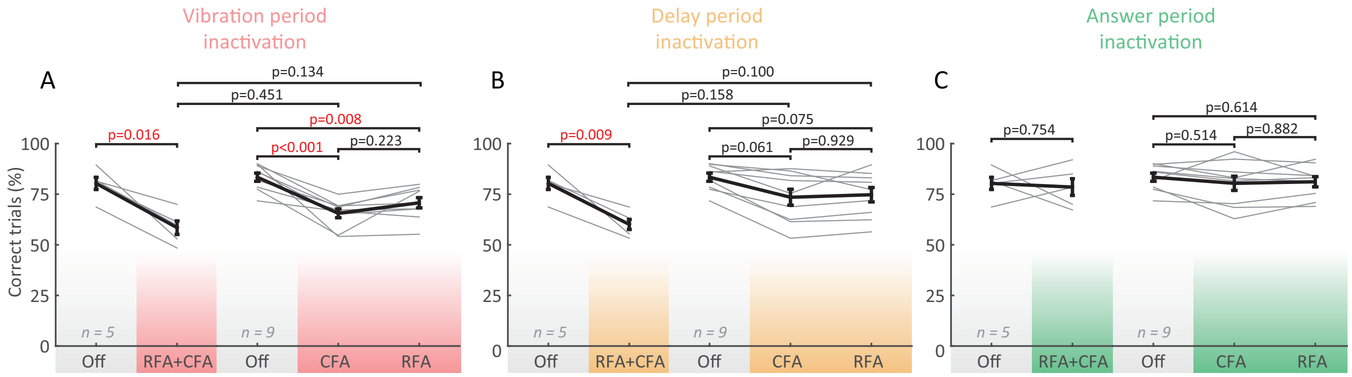


Comparison between the proportion of correct trials (among correct and incorrect trials) in control trials (e.g. Off, grey) and trials silenced on combined and single areas at different periods: vibration period (red, **A**), delay period (orange, **B**) and answer period (green, **C**). Black error bars represent the mean ± SEM of the proportion of correct trials (**A to C**). Fine grey lines: individual mouse performance.
